# Breast cancer in women 40–50 and over 70 years of age: the need for screening in Brazil

**DOI:** 10.1590/0100-3984.2025.0110

**Published:** 2026-04-23

**Authors:** Ivie Braga de Paula, Luciano Fernandes Chala, Linei Augusta Brolini Delle Urban, Almir Galvão Vieira Bitencourt, Tatiane Mendes Gonçalves de Oliveira, Henrique Lima Couto, Paula de Camargo Moraes, Beatriz Medicis Maranhão Miranda, Thaís Paiva Moraes, Ana Claudia Mendes Rodrigues Mussauer, Ellyete de Oliveira Canella, Selma di Pace Bauab, Ana Lucia Kefalás Oliveira, José Luis Esteves Francisco, Marcela Bisighelli Schaefer, Aline Dias Silva Guerrero Guimarães, Gustavo Machado Badan, João Emílio Peixoto, Rosangela Requi Jakubiak

**Affiliations:** 1 Casa Delas, Instituto Orizonti de Longevidade e Saúde, Belo Horizonte, MG, Brazil.; 2 Grupo Fleury, Belo Horizonte, MG, Brazil.; 3 Clinica Conrad, Belo Horizonte, MG, Brazil.; 4 Grupo Fleury Medicina e Saúde, São Paulo, SP, Brazil.; 5 Clínica de Diagnóstico Avançado por Imagem (DAPI), Curitiba, PR, Brazil.; 6 A.C.Camargo Câncer Center, São Paulo, SP, Brazil.; 7 Hospital das Clínicas da Faculdade de Medicina de Ribeirão Preto da USP, Ribeirão Preto, SP, Brazil.; 8 Redimama-Redimasto, Belo Horizonte, MG, Brazil.; 9 Grupo Dasa, São Paulo, SP, Brazil.; 10 Lucilo Maranhão Diagnósticos, Recife, PE, Brazil.; 11 Rede Mater Dei de Saúde, Belo Horizonte, MG, Brazil.; 12 Hospital São Vicente de Paulo, Rio de Janeiro, RJ, Brazil.; 13 Centro de Mama, Hospital Quinta D’Or, Rio de Janeiro, RJ, Brazil.; 14 Mama Imagem, São José do Rio Preto, SP, Brazil.; 15 Sabin Medicina Diagnóstica, Uberaba, MG, Brazil.; 16 Faculdade de Medicina de São José do Rio Preto, São José do Rio Preto, Brazil.; 17 SONITEC Medicina Diagnóstica, Florianópolis, SC, Brazil.; 18 Hospital Santa Catarina Paulista, São Paulo, SP, Brazil.; 19 Grupo Fleury, São Paulo, SP, Brazil.; 20 Instituto de Radioproteção e Dosimetria/CNEN, Rio de Janeiro, RJ, Brazil.

**Keywords:** Breast neoplasms, Mammography, Mass screening, Brazil, Middle aged, Aged

## Abstract

Breast cancer is the most common malignancy among women in Brazil and the leading cause of cancer-related death in the female population of the country. Despite advances in treatment and the implementation of mammography screening programs, mortality rates have continued to rise. This increase is particularly evident among women under 50 years of age and those 70 years of age or older, groups that account for a substantial proportion of diagnosed cases and registered deaths in the country. Until recently, these age groups were not included in the screening guide-lines established by the Brazilian National Ministry of Health, although consistent scientific evidence demonstrates significant benefits of early detection in these populations, including reduced mortality, a higher proportion of early-stage diagnoses, and less aggressive therapeutic interventions. This article analyzes epidemiological data, the socio-economic impacts of breast cancer, and the scientific evidence regarding mammography screening, discussing the outcomes and limitations of national programs in Brazil. On the basis of that analysis, we advocate for the revision and expansion of public breast cancer screening policies to include women 40–49 and ≤ 70 years of age, as an essential strategy to reduce mortality, improve clinical outcomes, and promote greater equity in access to diagnosis and treat-ment of breast cancer in Brazil.

## INTRODUCTION

Breast cancer is the most common type of cancer among women, representing a significant cause of morbidity and mortality, in Brazil and worldwide. Mammography can be performed on women of any age who present with clinical symptoms. However, obtaining periodic mammograms in women in the age range at risk for developing breast cancer who do not present with symptoms can reduce mortality from the disease. This benefit has been observed in the United States and in some European countries, in which there is effective quality control of the entire screening program and its results. Among women in those countries, breast cancer is no longer the leading cause of cancer death, although it continues to be the most prevalent type of cancer. Although public policies for mammography screening have been in effect in Brazil for more than two decades, mortality rates from the disease continue to rise, suggesting inefficiency in early detection. The number of breast cancer cases observed in women between 40 and 49 years of age is higher in Brazil than in high-income countries. In addition, tumors in women over 70 years of age are frequently diagnosed at an advanced stage. Nonetheless, both age groups long remained outside the scope of public policies for mam-mography screening. In the Brazilian *Sistema Único de Saúde* (SUS, Unified Health Care System), it was previously recommended that population-based mammography screening for breast cancer be performed once every two years in women 50–69 years of age. Recently, those recommendations were updated, expanding screening to include women 50–74 years of age and guaranteeing access to mammography on demand (in a joint decision with a health care professional, after guidance on the benefits and disadvantages of screening) for women 40–50 years of age. However, routine screening is still not recommended for those in the 40- to 50-year age group, and the recommended frequency of the examination continues to be biennial.

The aim of this article is to review the scientific, epidemiological, and economic evidence supporting the expansion of mammography screening to include women 40–49 and ≥ 70 years of age in Brazil. We address the direct impact of these measures on reducing mortality, improving clinical outcomes, and optimizing the use of health care resources.

### Incidence of and mortality associated with breast cancer worldwide

The incidence of breast cancer has increased world-wide in recent decades, with a growth rate of 0.5% per year between 2010 and 2019^**(^1^)**^. In high-income countries, the higher prevalence of breast cancer reflects greater exposure to reproductive, hormonal, and behavioral risk factors, mainly related to having had fewer children, advanced age at first pregnancy, shorter duration of breastfeeding, use of oral contraceptives, and use of hormone replacement therapy, as well as alcohol consumption, obesity, and a sedentary lifestyle^**(^1^)**^. Low-and middle-income countries, such as those in South America, Africa, and parts of Asia, which historically had a low incidence of breast cancer, have seen a significant increase in the number of cases in recent decades. This has been attributed to behavioral changes that approximate the risk factors observed in high-income countries, resulting in higher incidence rates^**(^1^)**^. However, the greatest reductions in mortality are observed in high-income countries that have created organized screening programs and provided broad access to health care. Whereas five-year survival rates exceed 90% in most high-income countries, the estimated mean for 12 sub-Saharan African countries in the 2008–2011 period was 66%, with estimates as low as 12% for Uganda^**(^1^)**^.

Despite the high survival rates in high-income countries, the risk of death has still been highest in patients with larger tumor size and grade, negative receptors, a greater number of positive lymph nodes, and tumors detected outside of screening^**(^2^)**^. These discrepancies are largely influenced by factors such as a lack of adequate health care infrastructure, scarcity of screening programs, and limited availability of advanced treatments. In addition, sociocultural barriers often prevent women in these regions from seeking or receiving timely diagnosis and treatment.

### Incidence of and mortality associated with breast cancer in Brazil

For the three-year period from 2023 to 2025, it is estimated that there will have been 73,610 new cases of breast cancer per year in Brazil, which corresponds to an estimated risk of 66.54 new cases for every 100,000 women^**(^3^)**^. According to data from the Oncology Brazil Panel^**(^4^)**^, there were 40,953 diagnoses in 2018 and 65,283 in 2023, representing a 59% increase in the number of cases in six years. Despite being the most common cancer among women in all regions of Brazil, the geographical distribution of breast cancer reveals significant heteroge-neity^**(^3^)**^, with the incidence (cases per 100,000 women) being highest in the southeast (84.46), followed by the south (71.44), central-west (57.28), northeast (52.20) and north (24.99).

The multicenter study designated Amazona I, involving patients at centers in various regions of Brazil, dem-onstrated that 44% of tumors have occurred in women under 50 years of age^**(^5^)**^. Therefore, at least one in three women diagnosed with breast cancer via the SUS would be outside the screening age range previously recommended by the Brazilian National Ministry of Health (50–69 years). These women were not offered the possibility of early diagnosis of the disease through mammography screening^**(^4^)**^. In the United States, only 17% of cancer cases occur in women under 50 years of age, whereas 83% occur in those 50 years of age or older^**(^1^)**^. Nevertheless, American medical societies and the U.S. Preventive Services Task Force have united in support of the recommendation for breast cancer screening to begin at 40 years of age, due to the increasing incidence of the disease in younger women^**(^6^)**^.

Another peculiarity of Brazil is the phenomenon of an increased incidence of breast cancer in patients under 40 years of age, a group in which tumors are more aggressive and larger at diagnosis^**(^7^)**^. A study involving patients treated between 2009 and 2020 showed an increase in the proportion of cases of the disease in the subgroup of women under 40 years of age, which increased from 7.9% in 2009 to 21.8% in 2020^**(^8^)**^.

Despite more than 20 years of population-based breast cancer screening in Brazil, the number of deaths from the disease continues to increase, having risen from 16,069 in 2016 to 20,165 in 2024^**(^9^)**^. According to epidemiological surveillance data for Brazil^**(^10^)**^, 22% of all breast cancer deaths occur in women under 50 years of age and 34% occur in women over 70, demonstrating the impact of the disease in those age groups. In countries with a very high human development index, less than 10% of deaths occur in women under 50 years of age^**(^11^)**^.

There are many factors that might be responsible for the difficulty in reducing breast cancer mortality in Brazil: the exclusion of major population subgroups, such as women 40–49 years of age and those over 69, from mammography screening recommendations by the SUS; low quality examinations having been observed via mammography quality certification programs; the low coverage rate of screening programs, especially within the SUS; and the absence of an organized screening program, which results in delays in scheduling appointments, performing examinations, releasing results, and consequently in access to surgical treatment, radiotherapy, and chemotherapy^**(^12^)**^. One study demonstrated that the mean time from clinical suspicion to diagnosis is 31.7 days for patients who pay out of pocket, compared with 68.9 days for those with health insurance and 93.4 days for those treated via the SUS. This disparity is observed in different regions of the country, with the greatest differences and worst outcomes being in the poorest regions^**(^12^)**^. As a result, tumors are often diagnosed at a more advanced stage, which is associated with an increased risk of recurrence and death, in those regions. The proportion of women diagnosed with stage III disease is 32.3% in Brazil, compared with 21.1% in the United States^**(^13^)**^.

### Results of breast cancer screening programs offered by public and private health care systems in Brazil

In Brazil, mammography coverage for the population of women 50–69 years of age is insufficient. Via the private health care system, 58.1% of women in that age group underwent mammography in the last two years, compared with 26.3% of women served by the SUS, whereas the ideal coverage is 70%^**(^14^)**^. According to a 2024 report from the Brazilian National Cancer Institute, the SUS is responsible for 49.5% of mammography coverage in the country. Therefore, most examinations are performed within the private sector, which accounts for up to 58.8% of coverage in the central-west region^**(^3^)**^. This highlights the importance of the private sector in breast cancer screening in the country.

Within the SUS, only 4.7% of breast cancer diagnoses are carcinoma *in situ*, whereas 41.2% are locally advanced or metastatic tumors, of which 76.4% are diagnosed in stage II, III, or IV and 64.5% are diagnosed in stage II or III**(^3^)**. In 2021, 52.0% of patients treated for breast cancer via the SUS were diagnosed with locally advanced stage III disease, compared with only 36.4% of those treated via a private health care plan^**(^15^)**^.

### Benefits of screening for breast cancer for women 40–49 years of age in Brazil

According to data for 2024 from the Brazilian Institute of Geography and Statistics^**(^16^)**^, 49.1% of women in Brazil are financially responsible for their households, comparable to the proportion of households in which a man is in that role. The median age of the female population in Brazil is 35 years^**(^16^)**^. In some states, such as Sergipe, Maranhão, and Rio de Janeiro, more women than men are the main providers in their households. Of the households in Brazil receiving financial aid from the federal government in 2022, 81.5% were headed by a woman^**(^17^)**^. Breast cancer diagnosed at an advanced stage in a woman who is the financial head of a household can impact an entire family that depends on her.

A prospective, randomized controlled study conducted in the United Kingdom, the Age Trial, showed a 25% reduction in the relative risk of death from breast cancer in the first 10 years of screening among women 39–49 years of age^**(^18^)**^. Despite the limited data available for this population, meta-analyses have demonstrated a 14–29% reduction in mortality in that age group when screening is implemented^**(19,20)**^. Another study, conducted in the municipality of Ipatinga, located in the Brazilian state of Minas Gerais, retrospectively evaluated mam-mography screening using the medical records of women 40–49 years of age^**(^21^)**^. The authors demonstrated that women who were not screened had later diagnoses and a five times higher mortality rate, with screening enabling earlier diagnoses even of biologically more aggressive tumors, as well as that women who underwent screening were less likely to undergo radical breast surgery and underwent fewer axillary lymphadenectomies.

It is estimated that 34% of the total years of life lost due to premature death from breast cancer are in women under 50 years of age, because, although they are not the age group with the highest percentage of deaths, they are younger women^**(^22^)**^. If all women ≤ 40 of age underwent mammography screening, the specific death rate from the disease could fall by up to 50%. In a meta-analysis on the benefits of mammography screening between 40 and 49 years of age, involving seven randomized studies initiated between 1963 and 1982, Smart et al.^**(^23^)**^ suggested that the benefits of mammography should be even greater, because the screening intervals were long (18–24 months) in those studies, in which mammography was performed at only one time point and new technologies such as digital mammography and tomosynthesis were not applied, given that those technologies have since greatly improved image quality and increased the sensitivity of the method.

In summary, the idea that mammography screening is associated with a reduction in the relative risk of death from breast cancer in women aged 40–49 years of age is supported by evidence considered to be of high quality and such reductions have been observed in numerous studies with various designs, including randomized and observational studies^**(^18^-^23^)**^.

### Benefits of screening for breast cancer in women over 70 years of age

One of the main risk factors for developing breast cancer is age. The risk of developing breast cancer is higher for women over 70 years of age than for women in the recommended age range for screening. Data from the United States show that the risk of developing invasive breast cancer is higher among women ≤ 70 years of age, for whom it is estimated to be 7.0%, compared with 3.5% for those between 60 and 69 years of age^**(^24^)**^. In 2022, there were 8.3 million women ≤ 70 years of age in Brazil. It is estimated that there will be 16.2 and 25.2 million women in this age group in the country in the years 2040 and 2060, respectively^**(^25^)**^.

The discrepancies regarding breast cancer screening in women ≤ 70 years of age stem from the fact that no randomized controlled studies have included women ≤ 75 years of age and only a few have included women between 70 and 74 years of age, although studies of the latter group have shown a 20% reduction in the risk of dying from breast cancer in those who underwent screening. In addition, the benefit in terms of survival does not become apparent for some time after diagnosis and treatment, therefore being related to life expectancy, which is determined by age itself and comorbidities^**(^26^)**^. In Brazil, it is estimated that the life expectancy for women at 70, 75, and 80 years of age is 15.8, 12.4, and 9.4 years, respectively^**(^25^)**^.

The best evidence on breast cancer screening in women ≥ 70 years of age comes from observational studies or mathematical models and supports screening such women^**(^27^-^29^)**^, showing greater sensitivity and specificity of mammography; a greater chance of diagnosis of a tumor in an early stage; a lower risk of dying from breast cancer; greater overall and disease-free survival; the possibility of less aggressive therapeutic options, which is very important in this age group; and a lower risk of false positives and radiation damage.

In Brazil, the proportion of women with advanced (stage III or IV) breast cancer is even higher in women ≥ 70 years of age. A study conducted by Rocha et al.^**(^30^)**^ showed that, among cases of breast cancer treated via the SUS in 2019, the proportion diagnosed at stage III or IV was 44.3% in women ≥ 70 years of age, compared with 40.8% (which is already high) in women 50–69 years of age. Those authors also showed that the risk of dying from breast cancer is significantly higher in women ≥ 70 years of age than in those 50–69 years of age, and that screening coverage in 2019 reached only 10.8% in the former group.

Physiological age alone does not reflect the complexity of aging and should not, by itself, define the termination of breast cancer screening. It is recommended that the screening be individualized, as well as that age, comorbidities, life expectancy, risks, benefits, tolerance to treatment, and the wishes of the patient all be taken into consideration.

### Risks of mammography screening

Mammography is the diagnostic imaging method used in breast cancer screening programs for asymptomatic women in many countries. However, because exposure to X-rays from the examination is a known risk factor for the induction of breast cancer itself^**(^31^)**^, it is important that the radiation doses produced by the X-ray equipment used in these programs are monitored and maintained under strict control by health authorities and existing mammography quality control programs in the country^**(^14^)**^. The dosimetric quantity associated with the risk of radiation-induced breast cancer is the mean glandular dose, which is the energy absorbed per unit mass of fibroglandular tissue (the most ra-diosensitive tissue of the breast), averaged for all of the fibroglandular tissue of the breast^**(^32^)**^. A recent study of 2,470 dose assessments between 2013 and 2024 showed that in Brazil, on average, the total mean glandular dose resulting from acquisition in the two views (craniocaudal and mediolateral oblique) is 3.40 mGy^**(^32^)**^. That dose level is equivalent to six weeks of exposure to natural environmental radiation^**(^33^)**^. Therefore, digital mammography presents a low risk of causing radiation-induced breast cancer in women in the screening age range. Other studies have shown that the number of lives saved due to early detection by screening programs is significantly greater than the number of mammography-induced cases of breast cancer^**(33,34)**^. Therefore, the number of lives saved by early detection may be on the order of 60 times greater than the number of deaths possibly caused by radiation exposure during mammography^**(^34^)**^.

Overdiagnosis refers to the detection of cancers that would never have become clinically evident or caused lifetime harm to the patient, even without treatment^**(^35^,^36^)**^. A meta-analysis by the EUROSCREEN group indicated that overdiagnosis rates are between 0% and 10%, with the highest rates being in older women. Under current standards, the overdiagnosis rate is less than 1% for women 40–49 years of age and less than 2% for those 50–59 years of age^**(^37^)**^. Conversely, older age and longer screening intervals lead to delays in the diagnosis of lethal tumors. A delay in discovering such tumors leads to underdiagnosis, with concomitant increases in anxiety, costs, treatment duration, morbidity, and mortality^**(^38^-^40^)**^. In reality, in Brazil, underdiagnosis is far more significant than overdiagnosis. Only 5% of patients treated via the SUS were diagnosed with carcinoma *in situ*, indicating a minimal rate of overdiagnosis. This indicates that, despite the importance of screening, the goal of early detection of cases is not being met effectively.

Approximately 10–12% of women undergoing screening by mammography are called back for follow-up studies, which can include additional tests or short-term monitoring. Some of those women (a minority) will need to undergo biopsy, and only one third of those who undergo biopsy will receive a diagnosis of breast cancer. The remainder will have benign lesions or findings related to tissue overlap and are considered false-positive cases^**(^41^)**^. On average, 9.6% of women who undergo screening need to return for additional tests^**(^42^)**^. Other studies indicate that the recall rate ranges from 9.6% to 11.6% for women of all ages^**(^42^-^44^)**^. Most patient recalls are resolved with additional imaging tests alone, without the need for biopsy. Biopsy, typically of the minimally invasive type, is indicated in less than 2% of such patients^**(^45^)**^. Although anxiety has been cited as a reason for postponing or reducing the frequency of mam-mography^**(^46^,^47^)**^, that concern does not take individual variability into consideration^**(^48^-^50^)**^. In addition, women diagnosed late with advanced cancer may experience greater anxiety and regret.

### Economic considerations

Annual mammography screening for women from 40 to 74 years of age, as recommended by medical societies, costs approximately three times more than biennial screening for women from 50 to 69 years of age, as previously proposed by the Brazilian National Ministry of Health^**(^51^)**^. However, making a more accurate estimate of costs is a much more complex task and requires taking into consideration the costs of treatment and the years of life lost due to breast cancer. Treatment becomes more expensive in parallel with increases in the stage of disease at diagnosis. In addition, the loss of economic productivity due to premature death, as well as the reduction in quality of life caused by more aggressive treatments, must be taken into account. Therefore, the costs of treatment and of the loss of productivity far exceed those of annual screening, even if the indirect value of lives saved is ignored^**(^52^,^53^)**^.

The cost of breast cancer treatment increases in parallel with the stage and age at diagnosis. In postmeno-pausal women in Brazil, treatment is 1.8 times more expensive for locally advanced (stage III) breast cancer than for early (stage I) breast cancer. In premenopausal women in the country, treatment for the former is 4.8 times more expensive than is treatment for the latter^**(^54^)**^. In addition, there has been an increase in the number of hospitalizations for breast cancer in all age groups in Brazil^**(^9^)**^. From 2018 to 2024, that number increased by 28.3% in women 40–49 years of age, by 29.4% in women 50–69 of age, and, most significantly, by 37.6% in women ≥ 70 years of age. Those data underscore the importance of early diagnosis in all age groups.

### Risk of not screening for breast cancer in certain age groups

It should be borne in mind that there is a risk in not screening for breast cancer. Advances in breast cancer treatment, although significant, have not overcome the disadvantage of being diagnosed with a tumor at an advanced stage. The adverse effects of annual mammography screening from 40 to 74 years of age and individualized screening from age 75 onwards are not lethal and are mainly related to the costs of operating the screening program. However, the adverse effect of not screening for breast cancer in women who are in the age range that accounts for more than half of deaths from the disease is an increase in the number of advanced diagnoses, which means more aggressive surgeries and chemotherapy with adverse effects, as well as in the number of deaths.

### Recommendations from Brazilian medical societies

In 2023, the Brazilian College of Radiology and Diagnostic Imaging, the Brazilian Society of Breast Specialists, and the Brazilian Federation of Gynecology and Obstetrics Associations jointly published an update to their recommendations for breast cancer screening, in the general and high-risk populations^**(^55^)**^, as detailed in Table 1. distinct screening recommendations in our country has always been a source of confusion within the medical community and among patients, contributing to the low coverage of screening programs and creating doubt about the benefits of early diagnosis.

**Table 1:** Breast cancer screening recommendations in the population at usual risk, by age, according to the Brazilian College of Radiology and Diagnostic Imaging, the Brazilian Society of Breast Specialists, and the Brazilian Federation of Gynecology and Obstetrics Associations.

Population	Recommendation
Women ≤ 39 years of age	Mammography screening is not recommended for the population at usual risk. Risk assessment using mathematical models is recommended from women ≥ 30 years of age, with the aim of identifying women at high risk who may benefit from differentiated screening strategies.
Women 40–74 years of age	Annual mammography screening, preferably with digital mammography or tomosynthesis if accessible and available, is recommended for all asymptomatic women.
Women ≥ 75 years of age	Individualized mammography screening is recommended, preferably with digital mammography or tomosynthesis if accessible and available, provided that there are no comorbidities that reduce life expectancy and that life expectancy is at least seven years.

Because of the profile of the population of Brazil, the inclusion of women 40–49 years of age and ≥ 70 years of age in mammography screening programs was one of the main points of change and was for decades a subject of debate between medical entities and the National Ministry of Health. According to medical societies in the country, women between 40 and 74 years of age should undergo breast cancer screening on an annual basis, preferably with digital mammography or tomosynthesis, if available, whereas women over 74 years of age should be screened on an individual basis. The recommendation of the National Ministry of Health that was in effect until September 2025 was that breast cancer screening with mammography should be performed every two years in women 50–69 years of age. The new screening recommendation from the National Ministry of Health continues to indicate biennial screening mammograms starting at age 50, but extends the age range to 74 years and guarantees on-demand access to mammograms for patients 40–49 years of age, without mandatory screen-ing every two years, as well as on-demand screening for

## FINAL CONSIDERATIONS

In women under 50 and over 70 years of age in Brazil, the incidence of and mortality rates associated with breast cancer increase every year, and women in those age groups account for the majority of cases of the disease in the country. The adoption of public policies that promote early detection is not only capable of reducing the rate of diagnosis at advanced stages but will also improve clinical outcomes and the quality of life of patients. In this context, the existence of two women over 74 years of age. This change represents an advance in mammography coverage and an opportunity for early diagnosis in women at the extremes of age, both younger and older.

From mammography to diagnosis and treatment, several steps are involved, such as identification of the suspicious lesion, acquisition of complementary views, preparation of the examination report, execution of image-guided ultrasound/biopsy, pathological analysis of the biopsy specimen, and referral for treatment. Further initiatives to improve the various steps of screening in Brazil are necessary to reduce the number of diagnoses in advanced stages and consequently reduce the mortality associated with the disease. Strategies to increase the coverage of screening programs and to increase patient adherence to screening recommendations are necessary and can be implemented not only by the government or medical entities but also by civil society. Strengthening quality mammography programs and helping patients with abnormal mammogram results navigate the system, thus allowing them access to subsequent exams, can reduce the time between identifying the abnormality and obtaining the histopathological diagnosis. Early diagnosis of breast cancer is a complex process, and quality control at all stages is essential. Thus, the treatment of the disease can be more efficient, less aggressive, and less costly, making early diagnosis the first step in reducing breast cancer mortality in Brazil.

## Data Availability

Not applicable
